# The prognostic significance of PD-L1 expression in patients with glioblastoma: A meta-analysis

**DOI:** 10.3389/fonc.2022.925560

**Published:** 2022-10-12

**Authors:** Xin Guo, Yuelin Zhang, Hengxing Jiao, Xingyu Miao

**Affiliations:** ^1^ Department of Neurosurgery, Shaanxi Provincial People's Hospital, Xi’an, China; ^2^ Department of Graduate Work, Hanguang Campus of Xi’an Medical University, Xi’an, China

**Keywords:** glioma, glioblastoma, PD-L1, CD274, B7-H1, prognostic glioma, a primary

## Abstract

**Background:**

Glioblastoma (GBM) is a malignant brain tumor associated with high morbidity and mortality rates with a poor prognosis. In recent years, studies on prognostic markers such as programmed death ligand 1 (PD-L1) have increased; however, their conclusions remain controversial. Here, relevant literature was reviewed and a meta-analysis was performed to clarify the correlation between PD-L1 expression and overall survival (OS) in GBM.

**Methods:**

The non-foundational literature on PD-L1 expression associated with OS in GBM up to February 2022 was searched in the PubMed, Metstr, Cochrane, and Web of Science databases. Literature was rigorously screened according to inclusion and exclusion criteria, the total hazard ratio (HR), and corresponding 95% confidence intervals (CIs).

**Results:**

Calculating the combined HR value and corresponding 95% CI of HR=1.124 (95% CI: 1.047–1.201, P=0.000, I2 (I-squared)=48.8%), it was shown that PD-L1 expression was significantly associated with low OS in GBM patients. Although I2 = 48.8% < 50%, to make the results more credible, in the cutoff values ≥10% subgroup HR=1.37 (95% CI: 1.07–1.67, P=0.000, I2 = 0%), which was also the result found in the first meta-analysis. In contrast, in the cutoff value ≥5% subgroup HR=1.14 (95% CI: 0.98–1.30, P=0.000, I2 = 59.8%) and in the cutoff value median PD-L1 expression levels subgroup HR=1.05 (95% CI: 0.92–1.18, P=0.000, I2 = 0%), indicating that PD-L1 expression was not associated with low OS in GBM. Furthermore, in four studies, we found no significant correlation between PD-L1 expression and the progression-free survival of GBM (HR=1.14, 95% CI:0.40–1.88, P=0.03, I2 = 29.3%).

**Conclusion:**

PD-L1 expression was significantly associated with low OS in GBM patients; however, this result needs to be interpreted with caution and requires a large, multicenter clinical study in patients with similar baseline data for further evaluation.

## Introduction

Glioma, a primary malignant central nervous system tumor, has high morbidity and mortality rates (1). The higher the pathological grade, the more malignant the tumor and the worse the prognosis; glioblastoma is considered the most malignant central nervous system tumor, with an overall 5-year survival rate of only 9.8% (2). The prognosis is very poor, even with a range of conventional treatments available such as surgical resection combined with postoperative radiotherapy (3). Fortunately, in recent years, treatments including the blockade of immune checkpoints (stimulatory or inhibitory factors that generate immune responses) and chimeric antigen receptor T-cell therapy have offered hope for patients (4, 5). Studies have shown that various malignancies are groundbreaking in this regard (6); for example, blocking programmed death 1 (PD-1)/programmed death ligand 1 (PD-L1) immune checkpoints can modify the prognosis of non-small cell lung cancer (NSCLC), gastric cancer, head and neck squamous cell carcinoma, and melanoma to some extent (7–11). With the successful treatment of melanoma and NSCLC with monoclonal antibodies against PD-1/PD-L1, immunotherapy may become a promising approach for the treatment of glioma (12, 13). Although PD-1/PD-L1 expression has been validated in GBM (14–27), the conclusions are controversial and require further investigation.

In this study, a meta-analysis based on previous studies was conducted to verify the correlation between high/positive PD-L1 expression and overall survival (OS) in GBM. Conclusions differing from previous studies were obtained, demonstrating the need for continued in-depth studies of PD-L1 to predict a prognosis in GBM.

## Materials and methods

### Study identification and data collection

This meta-analysis was conducted based on the recommendations and criteria developed by the Preferred Reporting Items for Systematic Reviews and Meta-Analyses (PRISMA) (28). By using keywords and free words (“GBM” or “glioblastoma”) and (“PD-L1,” “CD274,” or “B7-H1”) in the PubMed, Metstr (http://fmrs.metstr.com/index.aspx), Cochrane, and Web of Science data searches, all literature up to February 2022 was evaluated manually and screened for usable literature (G and J), and senior reviewers (Z) resolved any disputes therein. Finally, basic data from the included literature were extracted, such as the year of publication, first author, country, sample size, cutoff, material, assay method, staining pattern, presence of 1p/19q codeletion, O6 -methylguanine-DNA-methyltransferase (MGMT) methylation status, IDH mutation status, analysis methOS rate and 95% confidence interval (CI), progression-free survival (PFS), and 95% CI. The extraction of results was prioritized under multifactorial analysis and for the literature without a corresponding OS hazard ratio (HR) and 95% CI but with corresponding Kaplan–Meier curves. The interrupted point-taking method of Engauge Digitizer version 12.1 was used to extract the survival rates and transform them into HR and 95% CI.

### Inclusion and exclusion criteria

Inclusion and exclusion criteria were followed for article selection. The inclusion criteria were as follows: 1) the study investigated the prognostic value of PD-L1 expression in GBM; 2) literature was from the CGGA- and TCGA-published databases; 3) pathological findings were present confirming GBM in all patients with GBM; and 4) all patients were initially diagnosed with GBM. The exclusion criteria were as follows: 1) identical literature in different databases; 2) literature dealing with GBM recurrence; 3) similar meta-analyses; and 4) reviews, letters, and basic trials.

### Quality assessment

Two independent reviewers (G and J) assessed the quality of the included studies using the Newcastle–Ottawa Quality Assessment Scale (NOS) (29), which included three main entries (**Table 1**, 14–27). According to the relevant literature, an NOS score ≥5 was defined as high quality based on the literature (29).

**Table 1:** Basic characteristics of the included literature.

Author and year	Country	Index	Patients	Age mean ± SD	KPS	Material	Assay	Staining pattern	presence of 1p/19q codeletion				
Yawei Liu 2013	Denmark	Protein	GBM	58.53±10.13	NM	NM	IFC	NM	NM				
Nduom, k 2016	USA	Gene	GBM	NM	NM	TCGA	NM	NM	NM				
Berghoff 2014	Austria	Protein	GBM	58.81±10.43	≥70	FFPE	IHC	Membranous	NM				
Berghoff cohort 2014	Austria	Gene	GBM	NM	NM	TCGA	Agilent microarry	NM	NM				
Zeng, J. 2016	China	Protein	GBM	NM	NM	TMA	IHC	Membranes/Cytoplasm	NM				
Jiheun Han 2016	Korea	Protein	GBM	57.24±12.28	NM	TMA	IHC	membranous/cytoplasmic	NM				
Kyu 2018	Korea	Protein	GBM	58.18±12.09	NM	TMA/FFPE	IHC	Membranous/fibrillary	NM				
Arnon 2021	Denmark	Protein	GBM	64.15±8.08	NM	FFPE	IHC	NM	NM				
Dieter 2017	Germany	Protein	GBM	NM	NM	FFPE	IHC	NM	NM				
Dieter cohort 2017	Germany	Gene	GBM	NM	NM	TCGA	Agilent microarry	NM	NM				
Chia-Ing 2018	Italy	Protein	GBM	NM	≥70	ADCTA	IHC	patchy/diffuse fibrillary and geographic membraneous	NM				
Chia-Ing cohort 2018	Italy	Protein	GBM	NM	≥70	reference	IHC	patchy/diffuse fibrillary and geographic membraneous	NM				
Zheng 2016	China	Gene	GBM	NM	NM	CGGA	NM	NM	NM				
Zheng cohort 2016	China	Gene	GBM	NM	NM	TCGA	RNAseq data	NM	NM				
Drew Pratt 2018	USA	Protein	GBM	51.2±12.2	NM	TMA	IHC	NM	NM				
Drew Pratt cohort 2018	USA	Gene	GBM	NM	NM	TCGA	NM	NM	NM				
Zhiyuan Zhu 2020	China	Gene	GBM	NM	NM	TCGA	NM	NM	NM				
Lingrui Su 2020	China	Protein	GBM	NM	NM	NM	IHC	Membranous	NM				
Yasuo Takashima 2018	Japan	Gene	GBM	59.02±12.74	NM	TCGA	NM	NM	NM				
Author and year	MGMT methylation status	IDH mutation status	Cut off	Number	OS				PFS				NOS
					analysis method	HR	lci	uci	analysis method	HR	lci	uci
Yawei Liu 2013	NM	NM	percentage≥10%	17	NM	1.5	1.05	2.14	NM	NM	NM	NM	7
Nduom,k 2016	NM	NM	0.37	152	MA	1.52	1.03	2.25	NM	NM	NM	NM	7
Berghoff 2014	MGMT	NM	Percentage≥5%	117	NM	1.18	0.75	1.88	NM	NM	NM	NM	5
Berghoff cohort 2014	NM	NM	Median PD-L1 expression levels	446	MA	1.036	0.87	1.23	NM	NM	NM	NM	5
Zeng, J. 2016	NM	NM	Percentage≥5%	62	NM	1.32	0.96	1.82	NM	2.02	0.9	4.53	5
Jiheun Han 2016	NM	NM	Percentage≥5%	54	MA	4.958	1.557	15.79	UA	1.651	0.821	3.319	7
Kyu 2017	NM	IDH-1	Percentage≥5%	115	MA	1.204	0.584	2.485	NM	NM	NM	NM	6
Arnon 2021	MGMT	IDH wildtype	Median PD-L1 expression levels	163	MA	1.05	0.8	1.5	NM	NM	NM	NM	7
Dieter 2017	NM	IDH1/2 wildtype	NM	48	NM	0.973	0.5	2.0	NM	NM	NM	NM	7
Dieter cohort 2017	NM	NM	NM	467	NM	0.98	0.85	1.2	NM	NM	NM	NM	7
Chia-Ing 2018	MGMT	IDH-1	Percentage≥5%	27	MA	0.354	0.103	1.219	MA	0.528	0.178	1.563	7
Chia-Ing cohort 2018	MGMT	IDH-1	Percentage≥5%	20	MA	0.654	0.254	1.685	MA	1.435	0.498	4.137	7
Zheng 2016	NM	IDH1/2 mutations	NM	127	NM	2.2	1.49	3.24	NM	NM	NM	NM	6
Zheng cohort 2016	NM	IDH mutations	NM	160	NM	1.47	1.05	2.05	NM	NM	NM	NM	6
Drew Pratt 2018	NM	IDH wildtype	Percentage≥5%	125	NM	2.45	1.62	3.72	NM	NM	NM	NM	7
Drew Pratt cohort 2018	NM	IDH wildtype	Percentage≥5%	488	NM	1.19	1	1.42	NM	NM	NM	NM	6
Zhiyuan Zhu 2020	NM	NM	NM	150	MA	1.291	1.051	1.586	NM	NM	NM	NM	6
Lingrui Su 2020	NM	NM	percentage≥10%	47	NM	1.31	1	1.72	NM	NM	NM	NM	6
Yasuo Takashima 2018	NM	NM	Median PD-L1 expression levels	158	NM	1.07	0.88	1.29	NM	NM	NM	NM	6

IHC, immunohistochemistry; IFC, immunofluorescence histochemistry; FFPE, formalin-fixed paraffin-embedded; TMA, tissue microarray; TCGA, The Cancer Genome Atlas. UA,univariate analysis; MA, multivariate analysis; HR, hazard ratio; CI, confidence interval; NM, not mentioned; Cut off: Cut-off criterion for PD-L1 positive; KPS, Karnofsky PerformanceScore; PFS: progression-free survival.NOS: Selection (0–4 points): Representativeness of the exposed cohort, selection of the non-exposed cohort, ascertainment of exposure, demonstration that the outcome of interest was notpresent at start of study; comparability control for important factor (0–2 points); outcome (0–3 points): the assessment of the outcome, was follow-up long enough for outcomes to occur,adequacy of the follow-up of cohorts.

### Statistical analyses

The extracted HR values and 95% CIs were combined and displayed in deep forest plots, and the vertical line passing through one was defined as an invalid line that would suggest that PD-L1 expression does not predict a GBM prognosis. P<0.05 was defined as statistically significant. Furthermore, in the Cochrane Handbook criteria, heterogeneity is expressed by the Higgins I-squared (I2) statistic, and when I2 >50% indicates significant heterogeneity (30), a random-effects model was chosen to represent the final combined results; when it was <50%, a fixed-effects model was chosen. When there was significant heterogeneity, subgroup analysis, sensitivity analysis, and meta-regression were used to identify the sources of heterogeneity. Finally, funnel plots were used to detect the presence of publication bias, and when the funnel plots were asymmetric, Egger’s linear regression and Begg’s rank were used for further quantitative evaluation. The aforementioned data analyses were performed using Stata 16.0.

## Results

### Search results

**Figure 1:**
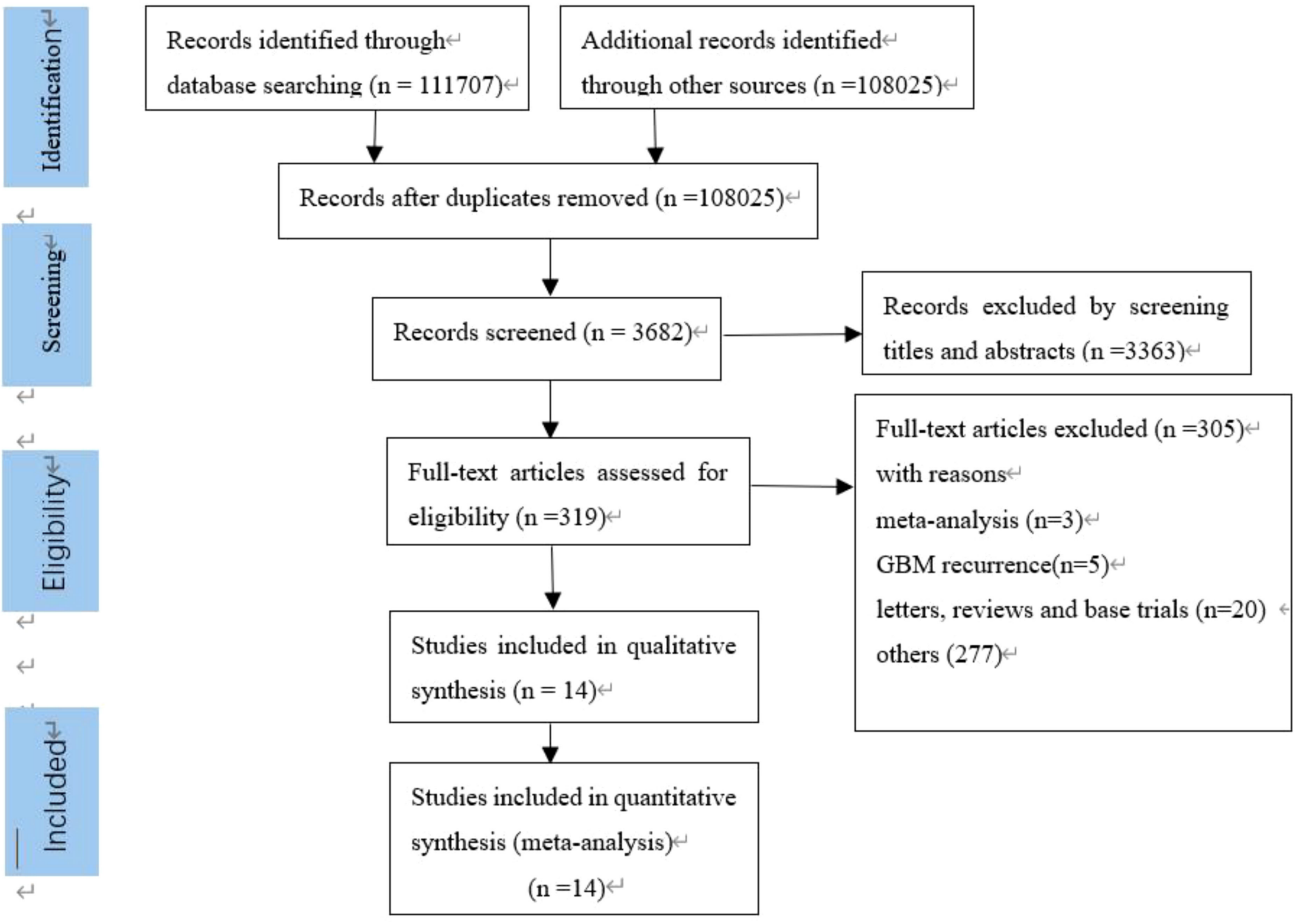
Selection process for the including studies.

A total of 319 publications were initially identified based on the search strategy. Of these, 305 were subsequently excluded for the following reasons: meta-analysis (n=3); GBM recurrence (5); letters, reviews, and base trials (20); and other (277), of which one was excluded for providing HR and 95% CI but the total number of patients for which HR was calculated was unclear (31); another did not provide HR and CI (32) (**Figure 1**). In total, 14 papers, containing 19 studies, were finally identified for inclusion (**Table 1**). The 19 studies included 2,943 patients; 8 studies assessed PD-L1 expression from gene expression, and the remaining 11 assessed PD-L1 expression from protein expression. A total of 10 cases showed PD-L1 expression detected by immunohistochemistry (IHC), and 1 case was detected by the immunofluorescence histochemistry (IFC) method. Of these, PFS values were present in four studies. The cutoff values for the percentage of PD-L1 expression–positive cells varied among the included studies, with eight studies having a cutoff value of ≥5%, five studies did not have a cutoff defined, three studies had a median PD-L1 expression level, two studies had a cutoff value of ≥10%, and one study had a cutoff defined as 0.37. Among the analysis methods, eight studies were multifactorial analyses and the others did not mention the analysis type.

### Correlation between overall survival, progression-free survival, and programmed death ligand 1 in GBM

**Figure 2:**
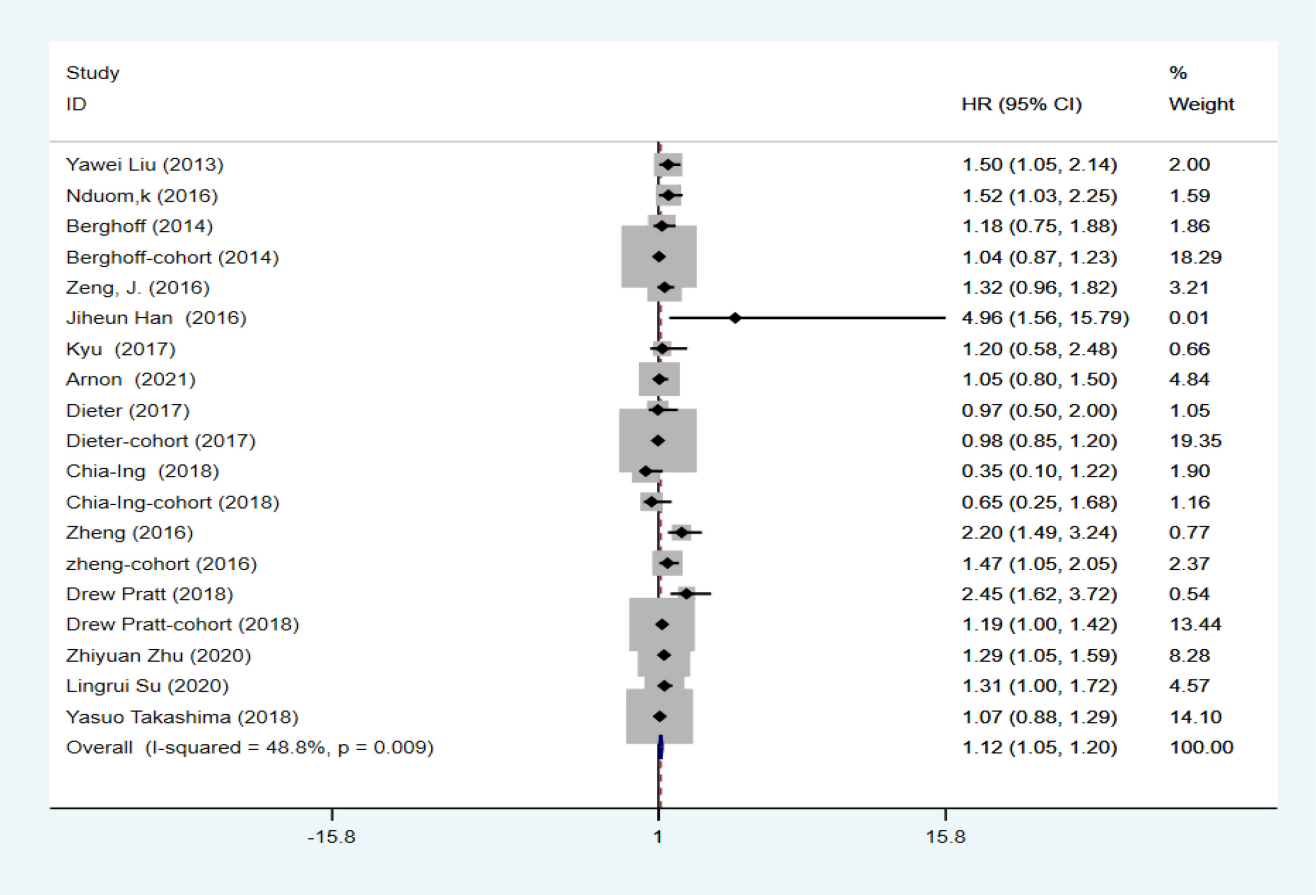
Association between PD-L1 expression and OS of GBM patients.

**Figure 3:**
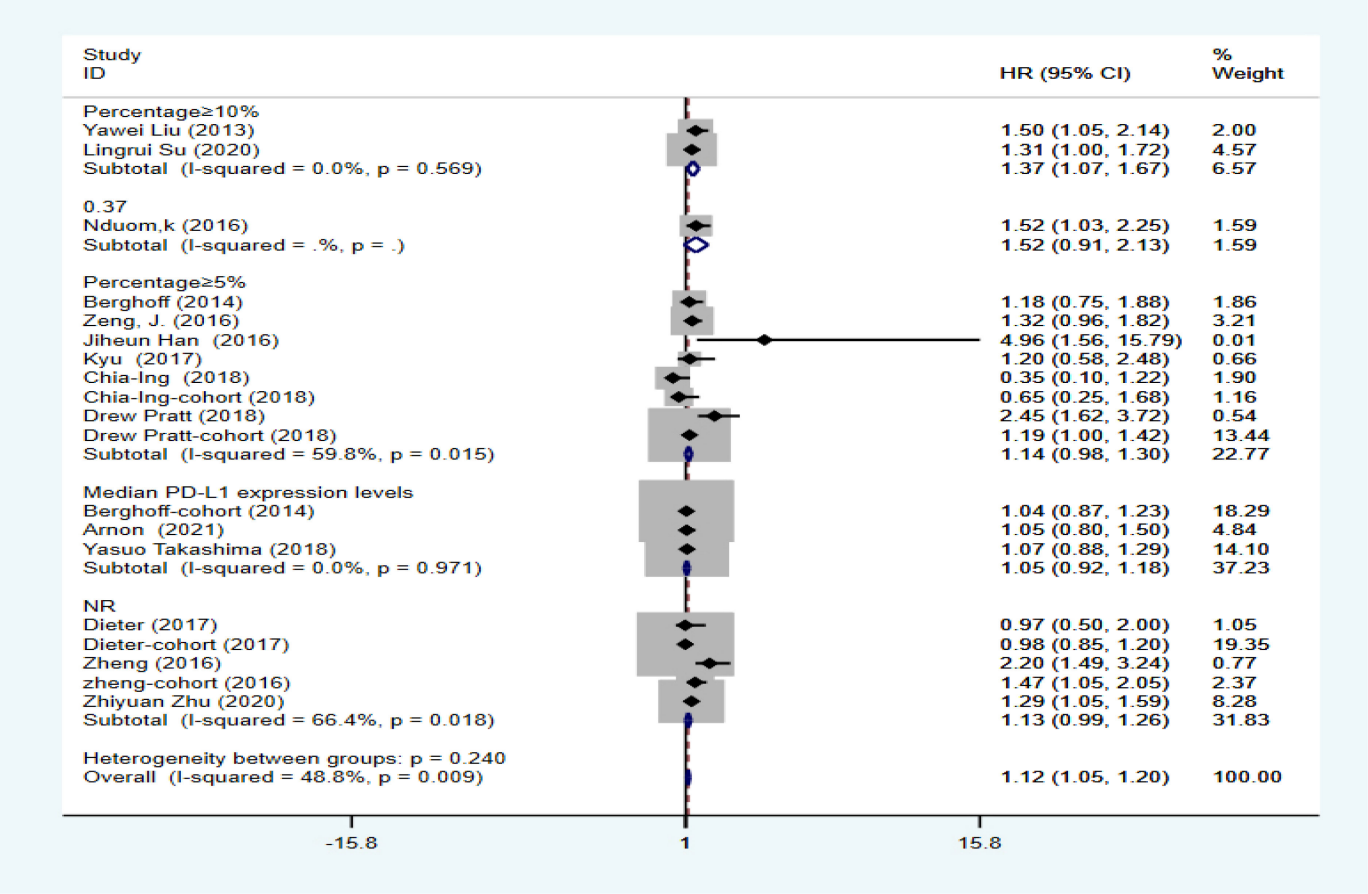
Analysis of subgroups with different cutoff values of PD-L1.

**Figure 4:**
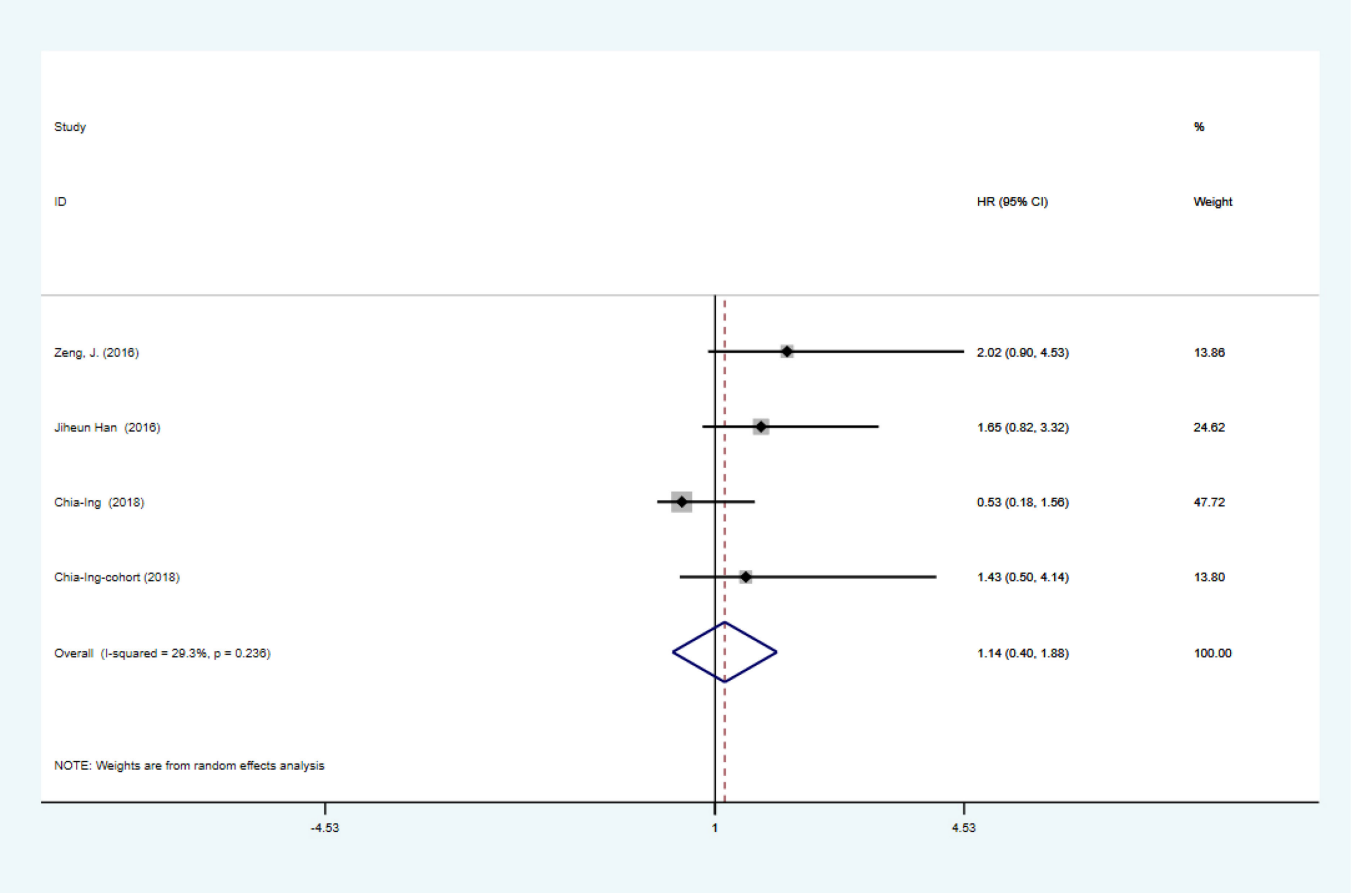
Association between PD-L1 expression and PFS of GBM patients.

The combined HR and corresponding 95% CI were calculated as HR=1.124 (95% CI: 1.047–1.201, P=0.000, I2 = 48.8%), indicating that PD-L1 expression was significantly associated with OS in GBM (**Figure 2**). Although I2 = 48.8% < 50%, to make the results more credible, in the cutoff values ≥10% subgroup HR=1.37 (95% CI: 1.07–1.67, P=0.000, I2 = 0%), which was also the result found in the first meta-analysis. In contrast, in the cutoff value ≥5% subgroup HR=1.14 (95% CI:0.98–1.30, P=0.000, I2 = 59.8%) and in the cutoff value median PD-L1 expression levels subgroup HR=1.05 (95% CI: 0.92–1.18, P=0.000, I2 = 0%), indicating that PD-L1 expression was not associated with low OS in GBM (**Figure 3**). Furthermore, in four studies, we found no significant correlation between PD-L1 expression and PFS in GBM (HR=1.14, 95% CI:0.40–1.88, P=0.03, I2 = 29.3%) (**Figure 4**).

### Heterogeneity and sensitivity analysis

**Figure 5:**
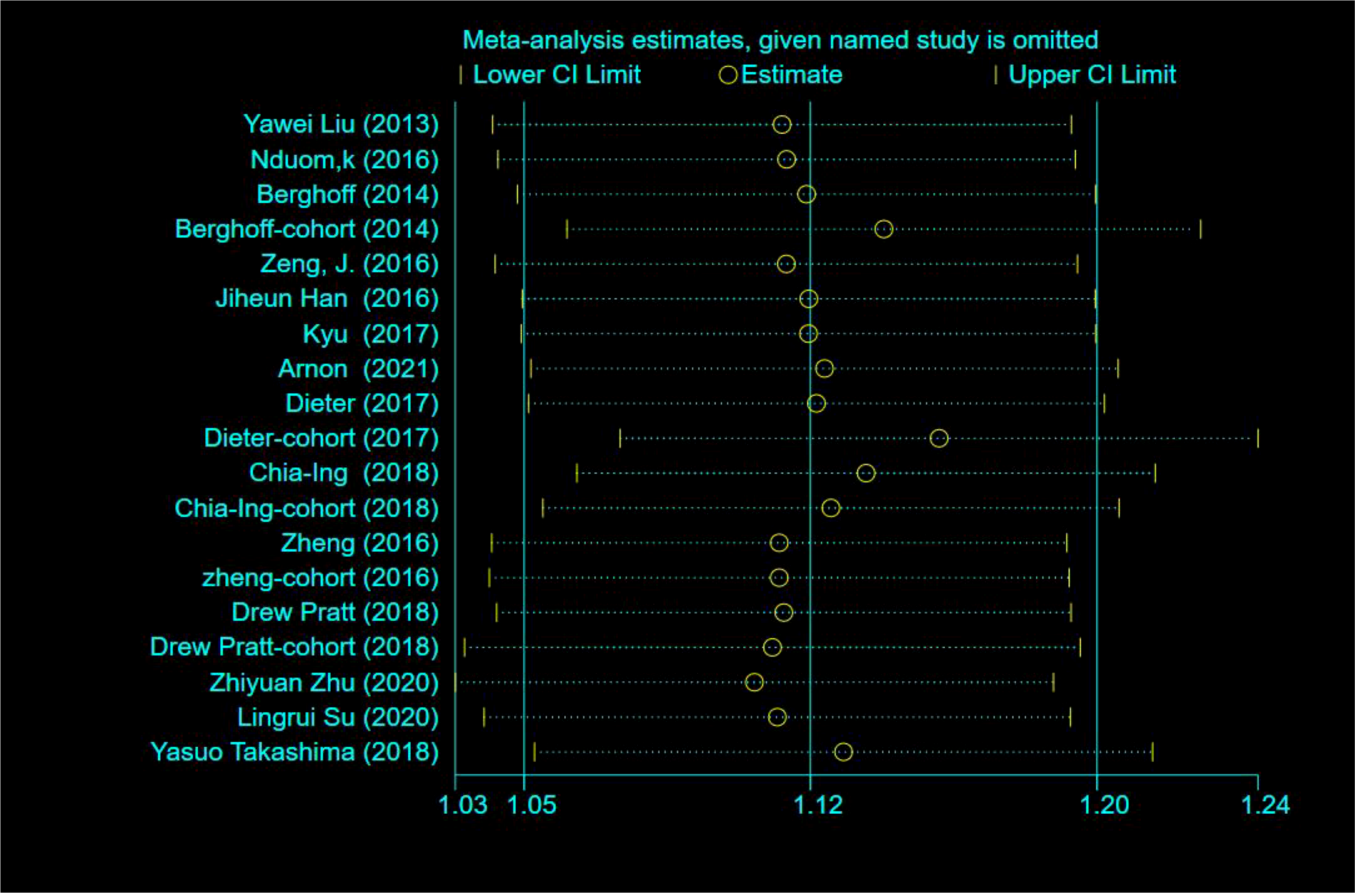
Sensitivity analysis.

**Figure 6:**
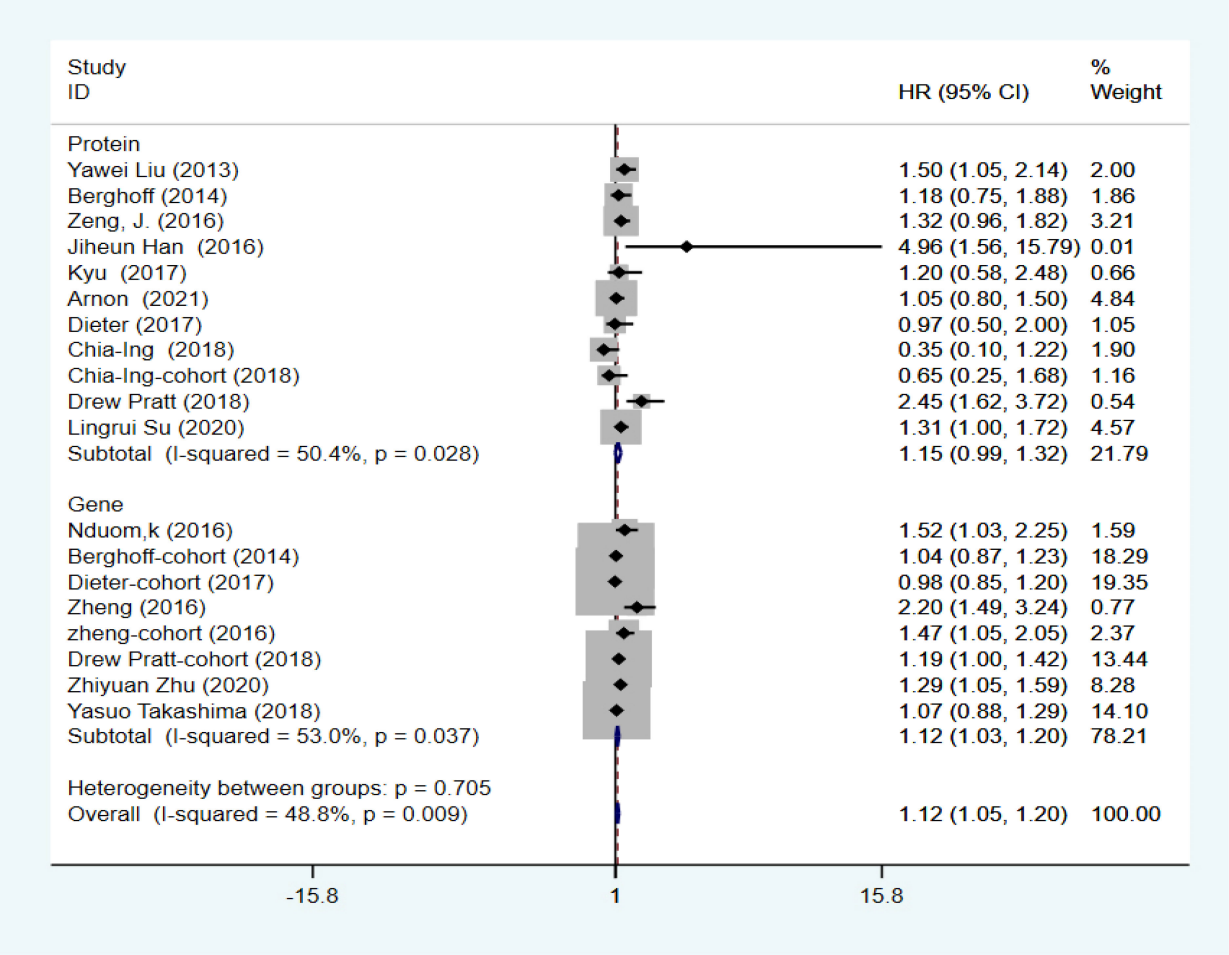
Subgroup analysis of PD-L1 expression in genes and proteins.

To further explore the sources of heterogeneity, the included literature data were analyzed using a sensitivity analysis; results showed that single studies were not associated with heterogeneity (**Figure 5**). Second, a subgroup analysis was performed based on gene and protein expression; results showed that in the gene expression subgroup HR=1.12 (95% CI: 1.03–1.20, P=0.000, I2 = 53.0%) and in the protein expression subgroup HR=1.15 (95% CI: 0.99–1.32, P=0.000, I2 = 50.4%), there were no reductions of heterogeneity (**Figure 6**); Meta-regression was used to test whether the country, year, age, Karnofsky Performance Score (KPS), MGMT methylation status, IDH mutation status, assay, and method of analysis affected heterogeneity. The results of the meta-regression showed that the inclusion of four variables (eight variables) resulted in a heterogeneity between the studies of Tau 2 = 0, which was 0.0303 less than the previous value of 0.0303 in **Figure 2**, implying that these factors could be used to explain 48.8% of the heterogeneity between studies (**Table 2**).

**Table 2:** Meta-regression.

Hr	Coef.	Std. Err.	t	P>|t|	[95% Conf. Interval]
Country	-.0463142	.1435912	-0.32	0.754	-.3662554 .273627
Year	-.0577189	.2200952	-0.26	0.798	-.5481216 .4326837
Age	.0080237	.1152203	0.07	0.946	-.2487031 .2647506
KPS	-.2289972	1.450993	-0.16	0.878	-3.46201 3.004016
MGMT	-.1435453	.5214006	-0.28	0.789	-1.305298 1.018208
IDH	.0567062	.2178329	0.26	0.800	-.4286556 .5420681
Assay	-.080288	.3354776	-0.24	0.816	-.8277787 .6672027
Analysis method	.0774851	.6897726	0.11	0.913	-1.459424 1.614394
_cons	3.97794	10.0847	0.39	0.702	-18.49216 26.44804

Meta-regression Number of obs = 19; REML estimate of between-study variance tau2 = 0; % residual variation due to heterogeneity I-squared_res = 0.00%; Proportion of between-study variance explained Adj R-squared = %; Joint test for all covariates Model F.

### Publication bias

Funnel plots were used to assess the presence of publication bias (**Appendix A Figures A1**); when the funnel plots were asymmetric, Egger and Begg tests were used for quantitative assessment (**Appendix: Figures A2**, **A3**). As shown in **Table 3**, P=0.18 > 0.05, 95% CI: –.5499226–2.712886, indicating the absence of publication bias in the included studies.

**Table 3:** Egger and Begg tests.

Std_Eff	Coef.	Std. Err.	t	P>|t|	[95% Conf. Interval]
Slope	.04155	.1131381	0.37	0.718	-.1971505 .2802505
Bias	1.081482	.7732451	1.40	0.180	-.5499226 2.712886

## Discussion

PD-L1 is expressed by a variety of cell types, including macrophages, T cells, B cells, and a subset of non-hematopoietic cell types such as vascular endothelial cells (33). Aberrant expression is present not only in glioma cell lines but also in other tissue specimens (34–36). To date, little is known about the mechanisms regulating PD-L1 expression, although one study suggested that it is regulated by two basic mechanisms: innate immune resistance–mediated structural expression and acquired immune resistance–mediated inducible expression (37). In tumor cells, interferon gamma (IFN-g-), which responds to an antitumor immune activity, is a major regulator of PD-L1; PD-L1 expression in tumors can also be activated by oncogenic mutations, such as the deletion of phosphatase and tensin homologue in gliomas (38–40).

PD-L1 expression has been observed in cancers such as NSCLC, melanoma, and colorectal cancer (41–43). In addition, PD-L1 expression has been found in gliomas, but the significance in predicting its expression in glioma patients remains controversial. For example, a study by Knudsen et al. (20) on 163 patients with glioblastoma showed that high median PD-L1 expression levels were not significantly related to prognosis, both under univariate and multifactorial analyses (univariate: HR=0.89, 95% CI: 0.66–1.23, P=0.58; multifactorial: HR=1.05, 95% CI: 0.8–1.5, P=0.8). In contrast, Han et al. (18) performed an immunohistochemical analysis of pathological sections from 54 glioma patients and defined at least 5% of cells detected by membrane staining as PD-L1 positive (without regard to staining intensity); high PD-L1 expression was related to gliomas and OS under multifactorial analysis (HR=4.958, 95% CI:1.557–15.79). Although there have been an increasing number of studies on the role of PD-L1 in predicting glioma prognosis in recent years, the conclusions remain mixed. Even though the index, patients, materials, assay, staining pattern, cutoff date, and analysis methods were the same as Han et al. (18), a study by Lee KS (19) showed that PD-L1 expression did not predict prognosis in glioma patients (HR=1.204,95% CI: 0.584–2.485, P=0.615).

Because of this inconsistency, this study reviewed and analyzed all the published literature on the role of PD-L1 expression in predicting OS in GBM patients. The results showed a correlation between PD-L1 expression and low OS in GBM patients. Further subgroup analysis showed that PD-L1 did not correlate with low OS in GBM patients in terms of protein expression, which was inconsistent with the findings of Wang H (44). Through discussion, it was found that the HR values included in this study were multifactorial, whereas the study by Wang et al. was univariate. In addition, this meta-analysis found a statistically significant heterogeneity of 0% (P=0.000) in the subgroup with a PD-L1 cutoff ≥10% for the first time, although further studies are needed to demonstrate this due to the paucity of literature. In the meta-regression, differences in the country, year, age, KPS, MGMT methylation status, IDH mutation status, assay, and method of analysis affected heterogeneity.

Fortunately, even though no immune checkpoint inhibitors have been approved for glioma (24), the efficacy of PD-1/PD-L1 inhibitors has been validated in preclinical glioma models; PD-1/PD-L1 inhibitors have been shown to restore antitumor T-cell activity and improve survival, which provides a theoretical basis for clinical trials (45–50). For example, in a study by Reiss SN (51), pembrolizumab prolonged PFS in some patients despite a low response rate. In addition, Lim (52) confirmed that nivolumab was well tolerated by newly diagnosed GBM patients, the incidence of adverse events was consistent with other reported neurological frequencies, and no deaths due to drug toxicity were reported; however, survival data require further follow-up. Therefore, there is a need to further investigate the relationship between PD-L1 expression and low OS in GBM patients. In addition, it has been shown that PD-L1 mRNA is expressed in all glioma grades and shows grade dependency (25, 53), possibly due to the association of expression with the vascular endothelial growth factor, matrix metalloproteinase 9, and KI-67. Further studies are needed to better understand these relationships.

Although the present study showed a correlation between PD-L1 expression and low OS in GBM patients, there are some limitations. First, although we analyzed the effect of the MGMT methylation status and IDH mutation status on heterogeneity, we could not further analyze the prognosis of PD-L1 expression in the MGMT methylation status or IDH mutation status only because the MGMT methylation status or IDH mutation status is not mentioned in part of the literature. Second, this study was retrospective and, as such, it was not possible to define GBM according to the latest WHO pathological classification (54), and only four studies mentioned PFS values. In addition, the impact of treatment on the prognosis of GBM patients could not be further explored because some treatment options were not mentioned. Finally, despite the low heterogeneity of the present study, PD-L1 in protein expression was not shown to be associated with low OS in GBM patients in the subgroup analysis, and despite the analysis of the reasons for this, further studies are needed to demonstrate the relationship between PD-L1 and the prognosis of GBM patients.

## Conclusions

In conclusion, despite the limitations of this study, a correlation was found between PD-L1 expression and poor OS in GBM. Statistically significant heterogeneity was found in the subgroup with a PD-L1 cutoff of ≥10% after pooled analysis, providing a theoretical basis for prospective clinical studies. Furthermore, meta-regression analysis demonstrated that the country, year, age, KPS, MGMT methylation status, IDH mutation status, assay, and method of analysis-affected heterogeneity were sources of heterogeneity. After controlling for these, the correlation between PD-L1 expression and low OS in GBM patients will become clearer, and interventions aimed at improving patient prognosis will become more defined.

## Author contributions

XG is responsible for article screening, writing and analysis; YZ selects the article direction and guides the article framework; HJ is responsible for article screening and data analysis; XM is responsible for article embellishment and guides article revision, submission and payment of page charges.

## Funding

This study was supported by a grant (No. 2022SF-166) from the Shaanxi Province Key R&D Program Projects.

## Conflict of interest

The authors declare that the content of the article was composed without any commercial or financial relationsh ip and without any conflict of interest.

## Publisher’s note

All claims expressed in this article are solely those of the authors and do not necessarily represent those of their affiliated organizations, or those of the publisher, the editors and the reviewers. Any product that may be evaluated in this article, or claim that may be made by its manufacturer, is not guaranteed or endorsed by the publisher.
