# Supplementary material for: The prognostic significance of PD-L1 expression in patients with glioblastoma: A meta-analysis
Source: Front Oncol. 2022 Oct 12;12:925560. doi: 10.3389/fonc.2022.925560 (PMC9596987; doi:10.3389/fonc.2022.925560)
Supplement: Supplementary file 1 [file DataSheet_1.pdf]

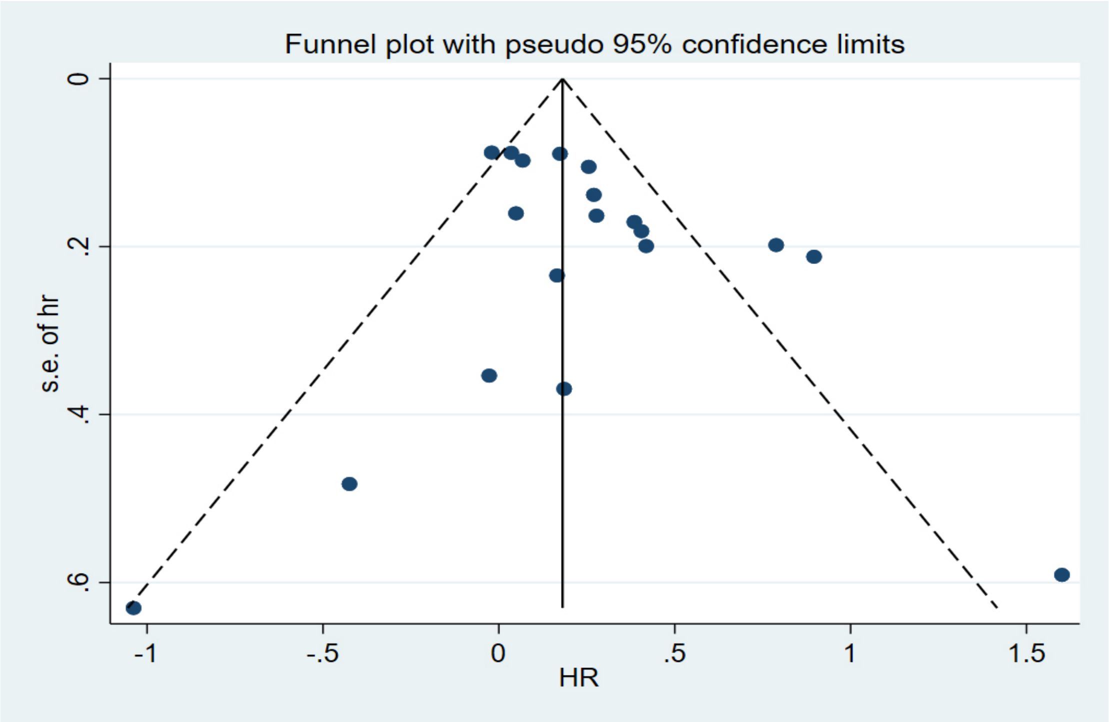

Appendix A Figures A1 Funnel Chart

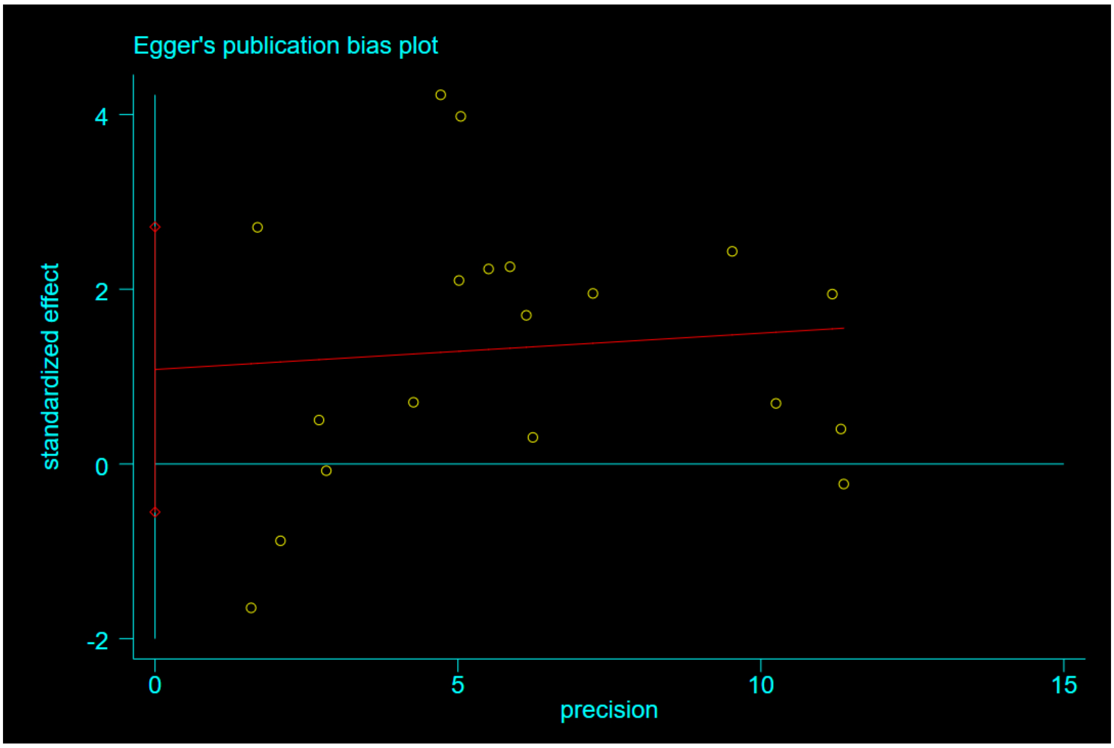

Appendix A Figures A2 Egger’s publication bias plot

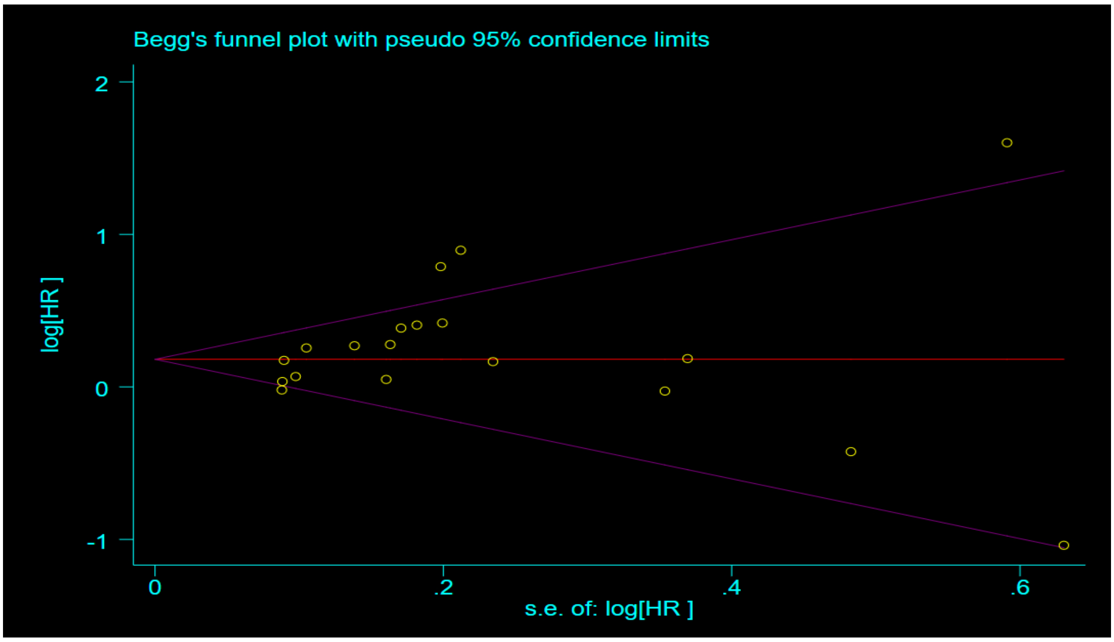

Appendix A Figures A3 Begg’s funnel plot with pseudo 95% confidence limits
